# Clinic Held for the Georgia Surgeons’ Club at Wesley Memorial Hospital, Nov. 5, 1913

**Published:** 1913-12

**Authors:** Willis Jones

**Affiliations:** Attending Surgeon to Wesley Memorial and Grady Hospitals


### CLINIC HELD FOR GEORGIA SURGEONS' CLUB AT WESLEY MEMORIAL HOSPITAL NOV. 5, 1913.

BY DR. WILLIS JONES, ATTENDING SURGEON TO WESLEY MEMORIAL AND GRADY HOSPITALS.

In selecting material for our clinic this morning, I have endeavored to get such cases as you meet in your routine daily practice. Have made no attempt to bring before you any of the rarer or more complicated surgical conditions.

The cases I have for operation today are such as will show you some of the varieties of hernia. Our first case gives the following history: A boy 14 years of age, occupation school, states that he was perfectly well up to one year ago; while plowing a rough field he was suddenly jerked forward by the handles of the plow shaft and immediately afterwards noticed a stinging sensation, accompanied by a fullness in the left groin; when he examined the groin he states that he detected, for the first time in his life, a mass about the size of a hen's egg; after a few minutes manipulation with his hands he was able to push this mass back into his abdominal cavity and he again started his work of plowing and after an hour or so noticed that the mass had returned. Being somewhat alarmed at the presence of this condition, which had never existed bebfore, he decided to quit his work and go home. He states that while walking home the mass returned several times and each time he was able to reduce it with his hands. He noticed, on going to bed at night, that the mass disappeared, but as soon as he was on his feet in the morning the mass always returned. There has never been any tenderness nor any inconvenience whatsoever from the presence of the mass. He thinks that during the past six months the size of the tumor has become somewhat larger than it first appeared to be one year ago.

From the history you will see that we have a typical reducible inguinal (scrotal) hernia. Looking over the statistics you will find that 75 to 80 per cent of all hernias are inguinal. In the male 93 to 95 per cent of hernias are inguinal. Inguinal hernia in the male occurs more frequently in the right side than on the left; this is due to the later descent of the testicle on the right side. Practically all of the hernias from the abdominal cavity make their appearance through the anatomical weak points, viz: the inguinal canal, femoral canal and around the umbilicus.

There are two general forms of inguinal hernia, the direct and the indirect, or oblique; the direct, I believe is usually due to direct violence, whereas the indirect, or oblique, I believe, is due to a weakened inguinal canal left by the remains of the vaginal process of peritoneum which follows the testicle down from the abdominal cavity through the inguinal canal into the scrotum, and which is never completely obliterated. I believe, furthermore, that the majority of indirect inguinal hernias in the adult as well as all of the infantile inguinal hernias, are due to a congenitally defective closure of this pouch of peritoneum. This case is a good illustration of this condition, because the boy states that his hernia was practically as large when he first noticed it as it is at the present time.

The operation which I do for the cure of inguinal hernia is usually the Bassini. I always do this operation unless the subject is one whose abdominal muscles are very poorly developed and where we would expect to find a very weak conjoined tendon; in this class of cases I prefer to do some modifications of the Bassini, or the Andrews operation.

Our patients are prepared for operation in the following way; upon admission to the Hospital they are given a tub bath and the hairy portions of the body are shaved on the evening of the day before the operation. On the morning of the operation the patient is carried to the operating room and the field of operation is painted with 50 per cent solution of tincture of iodine. This method of cleansing the skin has proven most efficacious in my hands and I have no regrets for having adopted it. One point in reference to this method I consider worthy of consideration; after the iodine has dried on the skin, if you endeavor to wash it off with alcohol, after the operation has been completed, I have found that the skin is much more likely to develop iodine dermatitis than if left alone; it seems that rendering the iodine soluble the second time adds very materially to its irritating qualities; this was first called to my attention by Dr. George Woolsey of New York City, about two years ago.

You will notice that I do not wear gloves while operating; my reasons for not doing so are as follows: First, the only advantage to be had from wearing the glove is practically a sterile hand. The disadvantages are as follows: First, the operator, when wearing gloves is more clumsy, his tactile sensations are materially impaired and when operating on the abdominal viscera he necessarily does more trauma with the gloved hand than with the hand without the glove; Second, considerably more time is required to operate with gloves than without; Third, where one uses a glove in every operation, the tendency to depend upon the glove for cleaniness rather than thorough cleansing of the hands before putting on the glove, grows with the individual, and should such be the case, if the glove is punctured during the operation it is a very easy matter to have the unclean contents from the finger of the glove drain into the field of the operation. I consider that these disadvantages far more than out-weigh the only advantage of being sure that the hand is clean. I always wear gloves in pus cases in order to keep my hands clean; I have my assistants wear them in all cases in order that I may know who to blame should an infection occur. From my observations, I get no more infections in my wounds than the operator who wears gloves in all cases.

I shall do a typical Bassini operation on this boy; I make my skin incision one and one-half inches above pouparts ligament extending from one inch above the internal ring down to the level of the external ring; this incision is carried down to the aponeurosis of the external oblique muscle; now the aponeurosis of the external oblique is slit up to a level of the inner ring, making the incision above the bulge produced by the cord lying beneath; this brings us down to the cord lying upon the conjoined tendon above the pouparts ligament below; we now very carefully separate the cord from these structures, pick it up with the fingers, cut through its fascial covering, also through the fibres of the cremaster muscle, careful to ligate every bleeding point; spread out the structure of the cord on the fingers of the left hand and look for the sac of the hernia; this usually is detected by its white, glistening appearance. With a piece of gauze, after the margin or edge of the sac has been separated, it is a very easy matter to separate the sac from the remaining structure of the cord. The vas deferens is usually the most difficult part to separate from the sac; you will notice that the sac lies somewhat above and to the inner side of the other structures of the cord; we carry on our separation upward until the sac is separated up as high as the internal ring; after this has been done, we cut through the fundus of the sack and insert the index finger of the left hand into the sac and force the contents of the sac, omentanum or intestine, back into the abdominal cavity, and while the finger is still in the sac, holding the abdominal contents back, a needle, threaded with No. 1 chromic gut is passed through the neck of the sac by the side of the finger and tied on the opposite side as the finger is gradually withdrawn from the neck of the sac, careful that no omentum or intestine is included in the ligature. After the neck of the sac has been securely tied, that portion of the sac dista to the ligature is cut and the ligated portion is dropped back into the abdominal cavity. We now pick up the cord and its structures and place a narrow strip of gauze under it to hold out of the field of operation, then we proceed to make a new floor for the canal by uniting the conjoined tendon of int. oblique and transversalis muscles to the inner lip of pouparts ligament; we place our sutures from above, downward about one-half inch apart; the first suture is placed as near as we can to the internal ring, so when tied, slight pressure is made on the cord; usually four to six sutures are required to suture the conjoined tendon to pouparts ligament. We now place the cord upon the new floor and unite the cut edges to the external oblique aponeurosis with interrupted No. 1 chromic ligatures. The skin is united with silk worm or metal clips: the wound is dressed with dry gauze; no spiker bandage is used in these cases. The wound is dressed and stitches removed the eighth to tenth day and the patient is allowed to go out of the hospital at the end of two weeks.

Our second case gives the following history: Female, age 30, married 12 years, has three children, the youngest, 5 years of age; after the birth of her first child 11 years ago, she suffered almost continuously from back ache and a sensation of pressure in her lower abdomen, up to February, 1911, when she had an operation for correction of a very marked retroversion of the uterus; since the operation she has felt perfectly well up to July, 1912, when her present trouble began. After lifting a heavy tub on the afternoon of July 5, 1912, she felt a sensation of something tearing loose in the lower right abdomen; the pains following this sensation were colicky in character; she noticed upon examination there was a fulness in the lower portion of the right groin which did not disappear until after she had gone to bed; she suffered no inconvenience during the night; the next morning after getting up and walking around for a few minutes her attention was again called to this uncomfortable sensation low down in the right groin. She immediately consulted her physician who informed her that she was ruptured. During the interval up to the present time, she has been unable to do any heavy work or lifting, for every time she would exert herself the mass in the groin would become much larger, but always disappear as soon as she assumed the recumbent position. You will note that the bulging in this case is below pouparts ligament as contrasted with the bulging in the boy upon whom we have just operated, where the bulging was above pouparts ligament. The mass in all femoral hernias makes its appearance below pouparts ligament and in inguinal hernias always above pouparts ligament. In the female, femoral hernia constitutes about 33 per cent of the hernias; inguinal about 50 per cent and the remaining hernias occur around the umbilicus. The hernial sac in this type of cases always comes down through the femoral canal on the inner side of the blood vessel. This type of hernia is never congenital and rarely occurs before the age of puberty and most frequent in those women who have borne children. We are never so sure of a cure in femoral hernia as we are in inguinal because we cannot close the femoral ring as securely as we can the inguinal ring. We make our incision one inch below pouparts ligament and parallel to, cutting down to the sac, which you can see here; we now caerfully separate the sac from the adjacent tissues, dissecting away all of the fat well up into the ring. We now cut through the fundus of the sac and we see omentum protruding; with the finger we push this omentum back into the abdominal cavity and with the finger still introduced into the neck of the sac we draw it down as far as possible and pass a No. 1 chromic gut through the neck of the sac just as close under pouparts ligament as we can; now, the ligature is tied securely around the neck of the sac and the distal portion of the sac is cut away; both ends of the ligature are left long, a needle is threaded on these ends and is passed up through the ring into the abdominal cavity, then up through the aponeurosis of the external oblique; this is done in an endeavor to draw the neck of the sac as far away from the abdominal opening of the femoral canal as possible; these ligatures are now tied and you will notice in the mouth of the canal an absence of the neck of the sac; this is the operation which is recommended by Mayo, Ochsner and others; they make no attempt to close the ring; I always feel that it is better to suture the under surface of pouparts ligament to the pectineal aponeurosis as high up as the pectineal eminence; this is practically the Bassini operation; so the operation that I have done so far on this case is a combination of that described by Mayo. Ochsner and others.

The skin is closed in the usual way with silk worm gut; the patients are kept in bed for two weeks after this operation and then allowed to go home, cautioning them to be careful and not strain from three to four months.

Our next case gives the following history: Male, age 55, machinist by occupation, was always well and strong up to the age of 30; in his thirtieth year he began to suffer from a sensation of heaviness in his left scrotum, he noticed that after standing for several hours the pain would radiate up through his left groin and around to his back; he also noticed that he was becoming more nervous and little things would worry him more than formerly; this uncomfortable sensation in his left scrotum gradually grew worse and ten years ago, in an endeavor to get relief, he was advised to put on a truss; he wore this truss for two years and he immediately noticed after putting the truss on that there was a marked increase in the size of the veins in the left scrotum; these veins finally became so large that he decided to discontinue the use of the truss; he felt more comfortable without the truss than with it; six years ago, after lifting a heavy piece of machinery, he experienced an uncomfortable sensation in the right lower abdomen; he paid very little attention to this for several weeks, until one day while coughing he noticed that each time the abdominal mucles were contracted as a result of his fits of coughing that there appeared a distinct mass in the right inguinal region; this condition has gradually enlarged and traveled downward, as he expresses it; six months ago he noticed a similar condition on the left side. Now you will observe that this patient has a double inguinal hernia, complicated by a very advanced varicocele on left side; you will also notice that his abdominal muscles are very poorly developed; this is the type of case that we do the posterior imbrication operation on as described by Andrews; the advantages of this operation over the Bassini is that we can make the floor of the canal more substantial by incorporating with the conjoined tendon the upper cut edge of the external oblique aponeurosis and this double layer of structures is sutured to the inner lip of pouparts ligament just as the conjoined tendon is in the Bassini operation. We then have the floor of the canal made up of conjoined tendon plus the upper layer of the aponeurosis of the external oblique; the roof of the canal is made by suturing the lower cut edge of the aponeurosis of the external oblique over and above the cord of the upper layer of the external oblique aponeurosis. We make our skin incision on the right side just as we did in our first operation this morning; we then slit up the aponeurosis of the external oblique, bringing us down to the cord which we isolate and pick up as in the regular Bassini operation; we isolate the sac, ligate, and drop back into the abdomen. Now, we elevate the cord with the piece of gauze and in placing our first suture, we include with the conjoined tendon the upper edge of the aponeurosis external oblique; then we go down and pick up the inner lip of pouparts ligament; this first ligature is placed as close to the exit of the cord from the internal ring as we can; by drawing on the suture, you will notice that we make slight pressure on the cord, not enough, however, to interfere with its circulation; the other sutures are placed at regular intervals of one-half inch distance from each other, each time including with the conjoined tendon the edge of external oblique aponeurosis. You will notice we have placed six sutures in this case. We begin and tie from above downward, drawing our ligatures just tight enough to approximate the tissues and not sufficiently tight to strangulate the incorporated portions; if we tie these ligatures too tightly, they cut through before the tissues have had time to unite and a separation results; this fact I consider responsible for a good number of returns after the operation for cure of inguinal hernia. Now we take the lower edge of the external oblique aponeurosis and carry up over the cord and suture to the aponeurosis of the external oblique with interrupted ligatures. Now the canal has been completed; we have the floor composed of conjoined tendon and external oblique aponeurosis and the roof made also of the lower portion of the external oblique aponeurosis; close the skin with silk worm gut; a gauze dressing is placed over the side, while we operate on the left.

Now on the left side we have the hernia, complicated by a very advanced varicocele; we proceed just exactly as we did on the right, exposing the cord, separating the sac, ligating, and returning to the abdominal cavity. Now we endeavor to cure the varicocele; we separate the vas deferens with its accompanying artery and vein from the other enlarged veins which compose the cord; we carry this separation well down to the scrotal part of the cord. We have separated here a distance of about two inches which is composed of markedly dilated veins; we tie off this separated mass of veins distally and proximally with No. 1 chromic gut, then we cut away the intervening portion; we bring the two cut ends together and tie around with the end of the same ligature that we used to ligate with primarily. We are very careful to see that the cut ends of the cord are securely tied, for should the ligature slip here, profuse hemorrhage would result. Notice now how the testicle has been drawn up since we have removed two inches of the dilated veins; as we have shortened the cord two inches, we have a vas deferens two inches longer than its accompanying cord. This is pushed back into the scrotum and the floor of the canal is made just as on the opposite side. The roof is also made as on the opposite side. We now close the skin in the usual manner and dress both sides with dry gauze. I do not curtail the scrotum in this patient, since it is very elastic and after a few days will contract up to correspond with that on the right side. The dressings on this patient will not be disturbed for 10 days, provided there is no infection; after that time the skin sutures are removed, patient kept in bed for 4 or 5 days longer and then allowed to leave the hospital, cautioning him not to lift or strain for several months.

319 Atlanta National Bank.

